# Efficacy of Anti-VEGF and Anti-EGFRs in Microsatellite Instable (MSI-H) Metastatic Colorectal Cancer in a Turkish Oncology Group (TOG) Cohort Study †

**DOI:** 10.3390/curroncol32110639

**Published:** 2025-11-14

**Authors:** İlknur Deliktaş Onur, Mutlu Doğan, Mehmet Akif Öztürk, Taha Koray Sahin, Murat Kiracı, Ahmet Melih Arslan, Eda Karapelit, Bahar Beliz Karaoğlan, Nargiz Majidova, Elif Şahin, Sabin Göktaş, Abdullah Sakin, Ali Oğul, Emine Türkmen, Kadriye Başkurt, Zeynep Yüksel Yaşar, Yakup Ergün, Esma Türkmen Bekmez, Şafak Yıldırım Dişli, Sinem Akbaş, Sema Türker, Ömer Dizdar, Öznur Bal, Tuğba Yavuzşen, Melek Karakurt, Arzu Hatime Yaşar, Tuğba Başoğlu, Faysal Dane, Şuayip Yalçın, Öztürk Ateş

**Affiliations:** 1 Dr. Abdurrahman Yurtaslan, Department of Medical Oncology, Ankara Oncology Education and Research Hospital, University of Health Sciences, 06200 Ankara, Turkey; 2 Department of Medical Oncology, Memorial Şişli Hospital, 34384 İstanbul, Turkey; 3 Department of Medical Oncology, University of Hacettepe, 06800 Ankara, Turkey; 4 Department of Medical Oncology, Bilkent City Hospital, University of Health Sciences, 06200 Ankara, Turkey; 5 Department of Medical Oncology, University of Dokuz Eylül, 35210 İzmir, Turkey; 6 Department of Medical Oncology, University of Necmettin Erbakan, 42310 Konya, Turkey; 7 Department of Medical Oncology, University of Ankara, 06050 Ankara, Turkey; 8 Department of Medical Oncology, University of Marmara, 34854 İstanbul, Turkey; 9 Department of Medical Oncology, Kocaeli City Hospital, University of Health Sciences, 41100 Kocaeli, Turkey; 10 Department of Medical Oncology, Kanuni Sultan Süleyman Education and Research Hospital, University of Health Sciences, 34668 İstanbul, Turkey; 11 Department of Medical Oncology, Bahçelievler Medipol Hospital, 34196 İstanbul, Turkey; 12 Department of Medical Oncology, Adana City Hospital, University of Health Sciences, 06200 Ankara, Turkey; 13 Department of Medical Oncology, University of Celal Bayar, 45047 Manisa, Turkey; 14 Department of Medical Oncology, Etlik City Hospital, University of Health Sciences, 06200 Ankara, Turkey; 15 Department of Medical Oncology, Kartal City Hospital, University of Health Sciences, 34668 İstanbul, Turkey; 16 Department of Medical Oncology, Bover Hospital, 21070 Diyarbakır, Turkey; 17 Department of Medical Oncology, Kayseri City Hospital, University of Health Sciences, 38039 Kayseri, Turkey; 18 Department of Medical Oncology, University of Koç, 34450 İstanbul, Turkey; 19 Department of Medical Oncology, VM Medical Park Maltepe Hospital, 34848 İstanbul, Turkey; 20 Department of Medical Oncology, Acıbadem Altunizade Hospital, 34662 İstanbul, Turkey

**Keywords:** dMMR colorectal cancer, MSI-H colorectal cancer, anti-EGFR, anti-VEGF

## Abstract

Background: Mismatch repair-deficient (dMMR)/microsatellite instability-high (MSI-H) colorectal tumors constitute 5% of metastatic colorectal cancer(mCRC). Immunotherapy is a new standard, but it is difficult to provide for all patients. 5-Flurouracil-based treatment with anti-EGFRs (cetuximab and panitumumab) in RAS/BRAF-wild or anti-VEGF (bevacizumab) is used in mCRC. Data is limited for the efficacy of anti-VEGF or anti-EGFRs in dMMR/MSI-H mCRC due to the small number of cases in the colorectal cancer population in trials. Aims: To evaluate prognostic factors in dMMR/MSI-H mCRC and compare progression-free survival time of patients receiving anti-VEGF and anti-EGFR combined with first-line 5FU-based therapy. Methods: Patients with metastatic dMMR/MSI-H colorectal cancer diagnosed between January 2015 and January 2023 were included in this cohort study. Progression-free survival times of patients treated with first-line therapy were compared. Prognostic factors associated with overall survival were investigated. Results: A total of 132 patients were included. Mutation rates were 35.6% (n:47) for RAS and 12.1% (n: 16) for BRAF (. Median progression-free survival (PFS) was 10.9 (95% CI: 9.2–12.6) months. Median overall survival (OS) was 44 months (95% CI: 26.23–63.03). 82 (62.1%) patients had primary tumor resection (PTR), 26 (19.7%) had PTR and metastasectomy. A total of 17 (12.8%) de novo mCRC patients had maximal cytoreductive surgery (MCS). A total of 14 (10.6%) patients had subsequent immunotherapy (IO). In multivariate analysis, RAS/BRAF mutation status, MCS, and subsequent IO are defined as prognostic factors for OS (*p* < 0.01, *p*: 0.022, and *p*: 0.005, respectively). No statistically significant difference (PFS, OS) was found in patients receiving first-line anti-VEGF or anti-EGFR therapy. Conclusions: dMMR/MSI-H mCRC is an entity with different tumor biology. We consider that dMMR/MSI-H mCRC patients with BRAF wild, MCS and subsequent IO have better outcomes with 1st line 5FU-based treatment with anti-VEGF/anti-EGFRs.

## Simple Summary

Colorectal cancer is a widespread health problem at present. Despite recent advances in metastatic colorectal cancer, median survival remains low. Microsatellite instability-high (MSI-H) colorectal cancer accounts for approximately 5% of all metastatic colorectal cancer patients and has a different tumor biology than other colorectal cancer patients. In our study, we evaluated the efficacy of commonly used targeted therapies in this subgroup of metastatic colorectal cancer patients. While we did not find a statistically significant difference between targeted therapies, we found that progression-free survival was numerically higher with bevacizumab. Furthermore, we found that BRAF mutation, maximal cytoreduction, and the use of second-line and subsequent immunotherapy were associated with prognosis.

## 1. Introduction

Colorectal cancer (CRC) is a common malignancy all around the world, with a higher incidence and mortality rate in males [1,2]. The regional incidence of CRC varies globally due to the geographical differences in terms of diet and environmental factors, besides differences in screening programs as well [3]. Most of CRC occurs sporadically. A total of 25 percent of the patients have a family history, while only 5–6% of CRC patients with a family history have hereditary backbone as inherited mutations in major CRC genes [4]. However, the accumulation of both genetic mutations and epigenetic modifications of several genes may cause CRC in the remaining part of cases [4].

DNA mismatch repair defects may contribute to carcinogenesis and/or progression in many solid tumors, including CRC. Mismatch repair-deficient (dMMR)/microsatellite instability-high (MSI-H) CRC tumors constitute 15% of all cases. Only 5% of metastatic CRC (mCRC) patients present with dMMR/MSI-H tumors [5]. DNA mismatch repair deficiency (dMMR) leads to microsatellite instability (MSI) with genomic alterations. Expression loss in MLH1, MSH2, MSH6, and PMS2 genes results in a deficiency in protein products of these genes, leading to the impaired detection of mismatched and unpaired bases [5]. dMMR/MSI-H CRC cases are often located in the proximal colon. They frequently have a greater mucinous component, higher lymphocytic infiltration, and are more often poorly differentiated. Although tumors in Lynch syndrome tend to be poorly differentiated, the presence of MSI mitigates the adverse prognostic impact of this feature, and in fact, MSI-H tumors are associated with longer survival in both Lynch syndrome and sporadic cases, for unclear reasons [6,7,8].

Metastatic dMMR/MSI-H CRC patients with somatic BRAF mutations have a worse prognosis and less chemosensitivity [9]. BRAF V600E mutation is associated with MLH1 promoter hypermethylation leading to deficiency in MLH1 and PMS2 proteins, which is the most common cause of the dMMR/MSI-H phenotype in patients without Lynch syndrome [10].

Immunotherapy has been shown to have better outcomes in dMMR/MSI-H cancer patients, including CRC [11]. So, it has become a new standard of care for these patients in recent years. On the other hand, it is not so easy to apply immunotherapy to all patients, especially in low-to-middle-income countries. We have used 5-flurouracil-based chemotherapy in mCRC for decades. It has been shown that the addition of targeted therapy, including epidermal growth factor receptor (EGFR) inhibitors, such as anti-EGFR monoclonal antibodies (panitumumab and cetuximab) and 5-flurouracil-based chemotherapy, in pan-Ras/BRAF wild-type patients with mCRC increases overall survival [12,13]. A bevacizumab, an anti-vascular endothelial growth factor (VEGF) monoclonal antibody, and chemotherapy combination has also been reported to have efficacy in mCRC patients, regardless of Ras/BRAF mutation status [14,15]. However, we have less data for the outcomes of targeted therapy, including anti-EGFRs (cetuximab, panitumumab) and anti-VEGFs (bevacizumab), in metastatic dMMR/MSI-H CRC due to the smaller proportion of these cases in the whole CRC population in clinical trials. We consider that there is a need for the enlightenment of the role of anti-EGFRs and bevacizumab in dMMR/MSI-H mCRC patients.

The aim of this study was to determine prognostic factors in patients with MSI-H mCRC and to compare progression-free survival (PFS) times in patients who received targeted therapy (anti-EGFR and anti-VEGF) combined with 5-FU-based chemotherapy in first-line treatment.

## 2. Patients and Methods

### 2.1. Study Population

This multicenter cohort study was conducted retrospectively. Patients aged ≥18 years diagnosed with dMMR/MSI-H mCRC between January 2015 and January 2023 were included in the study. Hospital registry databases and file documents were used to assess demographic and clinicopathological characteristics, treatment modalities, and outcomes. The study included dMMR/MSI-H colorectal cancer patients presenting with both de novo metastatic and recurrent disease. All patients had received 5-flurouracil-based chemotherapy as a first-line treatment for metastatic disease. Patients who could not receive treatment for any reason or who received immunotherapy as a first-line treatment were excluded. Patients with insufficient data from their files were also excluded.

### 2.2. Variable Measurement and Definition

Age, gender, ECOG-performance status (ECOG-PS), date of diagnosis, comorbidities, presence of secondary malignancy, tumor location, MSI status, and K-Ras/N-Ras/BRAF mutation status were recorded. In this study, dMMR/MSI-H status was assessed by immunohistochemistry at all centers, and patients with loss of staining in at least two of the MLH1-MSH2-MS6-PMS2 genes were considered dMMR/MSI-H. All treatment modalities were recorded in detail. Patients who underwent metastasectomy and received only chemotherapy (without targeted therapy) after surgery were excluded from the statistical analysis comparing PFS with patients receiving targeted therapy as a first-line metastatic disease. Overall survival (OS) was defined as the time from diagnosis to death or, for patients still alive, to the last visit. For de novo metastatic patients, OS was defined as the time from diagnosis to death, while for metachronous metastatic patients, OS was defined as the time from the date of metastasis to the date of death. PFS was defined as the time from diagnosis to progression with targeted therapy versus first-line systemic therapy. Survival data were last updated in May 2024.

The primary endpoint of this study was to compare PFS in patients with dMMR/MSI-H mCRC who received first-line anti-VEGF and anti-EGFR therapy. We also examined prognostic factors associated with PFS and OS in dMMR/MSI-H mCRC patients.

The manuscript follows the STROBE reporting guidelines [16].

### 2.3. Statistical Analysis

All analyses were performed using the SPSS 23.0 program. In the descriptive statistics of the study, continuous variables were used as mean (±standard deviation) and median (range); categorical variables were presented as frequency (percentage). Chi-square or Fisher’s Exact test was used to compare the categorical variables of two independent groups. The independent sample *t*-test and Mann–Whitney U test were used to compare parametric and non-parametric data, respectively. PFS1 and OS of patients receiving anti-VEGF and anti-EGFR were estimated with the Kaplan–Meier method and compared with the log-rank test. Univariate and multivariable logistic regression models were applied to evaluate factors predicting survival. A logistic regression model was created with variables with a *p*-value of <0.05, and independent factors predicting overall survival were identified. *p* < 0.05 was accepted as statistically significant

## 3. Results

Data from a total of 148 patients were reviewed. A total of 132 patients were included in the study. Sixteen patients who received immunotherapy as first-line treatment for metastatic disease were excluded from the study. The median age of the patients was 60 (23–82) years: 56 (43.2%) were female, and 67 (50.8) were male. In total, 81 (61.4%) patients had right colon, 33 (25%) had left colon, and 18 (13.6%) had rectum localized tumors. A total of 72 (54.5%) patients had ‘de novo’ mCRC, while 60 (45.5%) patients had recurrent disease. All recurrent CRC patients had distant metastasis at analysis. Forty-seven (35.6%) patients were Ras mutant, and sixteen (12.1%) patients were BRAF mutant. Fifteen (11.4%) of the patients were diagnosed with Lynch syndrome. The secondary malignancy rate was 12.1% (n: 16). Nine (56.2%) patients with secondary malignancy were diagnosed with Lynch syndrome (Table 1).

Primary tumor resection was performed in 82 (62.1%) patients. Sixty-one (46.2%) recurrent patients had surgery at diagnosis when they had nonmetastatic disease, while twenty-seven (20.4%) had primary tumor resection despite ‘de novo’ metastatic CRC at diagnosis. Maximal cytoreductive surgery (MCS) was considered when both primary tumor resection and metastectomy were performed, and the tumor could not be resected to R0. MCS was performed in 26 (19.7%) patients. A total of 17 (12.8%) patients with ‘de novo’ metastatic disease had undergone maximal cytoreductive surgery. Nine (6.8%) of the patients had a metastasectomy in case of recurrence. Systemic treatment was given to 29 (22%) patients after metastasectomy with curative intent. Forty out of one hundred thirty-two patients received maintenance treatment following first-line treatment, and ninety-two did not receive maintenance treatment. Eight of the patients who received maintenance treatment received only capecitabine. Thirteen patients received cetuximab in combination with 5-fu/capecitabine. Nineteen patients received bevacizumab together with 5-fu/capecitabine.

Median PFS with first-line systemic treatment was 10.9 (95% CI: 9.2–12.6) months. There was no significant relationship between PFS and patient characteristics, such as age, gender comorbidities, Ras/BRAF mutation status, metastatic sites, whether maximal cytoreduction was performed or not, and oxaliplatin- or irinotecan-based chemotherapy as a chemotherapy backbone with targeted treatment (*p*: 0.60, *p*: 0.55, *p*: 0.99, *p*: 0.53, *p*: 0.48, *p*: 0.89, *p*: 0.36, respectively). In subgroup analysis, the patients with left colon localization, BRAF wild type tumor, and maintenance treatment had significantly better PFS (*p*: 0.045, *p*: 0.037, *p*: 0.007, respectively) (Table 2). The median PFS was 9.3 (95% CI: 7.9–10.7) months in the right colon tumors, while it was 9.6 (95% CI: 5.8–13.4) months for rectum and 12.5 (95% CI: 9.1–15.8) months for the left colon-localized ones. When evaluated according to BRAF mutation analysis, median PFS in BRAF wild patients was 11.5 (95% CI: 6.5–16.5) months, while PFS in BRAF mutant patients was 8.9 (95% CI: 5.7–12.0) months. PFS was statistically significantly lower for the patients with BRAF mutation (*p*: 0.037). Median PFS for the patients using targeted therapy (anti-VEGF or anti-EGFR) was 12.5 (95% CI: 10.0–14.9) months, and 7.9 (95% CI: 4.6–11.3) months for the others without targeted therapy (*p*: 0.085). Anti-EGFRs were given to the patients with pan-RAS/BRAF wild-type tumors. There was no significant difference in PFS according to the type of targeted treatment, in spite of a numerically higher PFS for bevacizumab. The median PFS was 13.4 (95% CI: 9.7–17.1) months in patients receiving bevacizumab, 12.5 (95% CI: 10.2–14.7) months in patients receiving cetuximab, and 11.3 (95% CI: 2.7–19.9) months in patients receiving panitumumab.

The patients who had maintenance treatment had significantly longer PFS. Median PFS was 14 (95% CI: 12.4–15.5) months versus 8.7 (95% CI: 7.1–10.3) months (*p*: 0.007).

**Table 1:** Baseline characteristics of the patients.

		N (%)
		132 (100)
Age (median and range) in years		60 (23–82)
Sex		
	Female	56 (43.2)
	Male	67 (50.8)
Comorbid disease		
	Yes	62 (45.5)
	No	60 (47.0)
ECOG PS		
	0–1	124 (93.9)
	≥2	8 (6.1)
Secondary Malignancy		
	Yes	16 (12.1)
	No	106 (80.3)
Lynch Syndrome		
	Yes	15 (11.4)
	No	34 (25.8)
	Missing data	83 (62.8)
Primary tumor location		
	Right *	81 (61.4)
	Left **	33 (25)
	Rectum	18 (13.6)
Advanced stage		
	De novo metastatic	72 (54.5)
	Recurrence disease	60 (45.5)
MLH1		
	Wild	54 (40.9)
	Mutant	78 (59.1)
MSH2		
	Wild	74 (56.1)
	Mutant	58 (43.9)
MSH6		
	Wild	75 (56.8)
	Mutant	55 (41.7)
PMS2		
	Wild	61 (46.2)
	Mutant	68 (51.5)
Pan-Ras mutation		
	Wild	80 (60.0)
	Mutant	47 (35.6)
BRAF mutation		
	Wild	116 (87.9)
	Mutant	16 (12.1)
Surgery		
	No	24 (18.2)
	Primary tumor	82 (62.1)
	Primary tumor and metastasis	26 (19.7)
Systemic treatment after metastasectomy		
	No	98 (74.2)
	Yes	29 (22.0)
First-Line Therapy		
	Xelox ^&^ + bevacizumab	6 (4.5)
	Folfox ^&&^	32 (24.2)
	Folfox ^&&^ + bevacizumab	38 (28.7)
	Folfox ^&&^ + cetuximab/panitumumab	2015.1)
	Folfiri ^+^	2 (1.5)
	Folfiri ^+^ + bevacizumab	28 (21.2)
	Folfiri ^+^ + cetuximab/panitumumab	6 (4.5)
Immunotherapy as subsequent line		
	Yes	14 (10.6)
	No	118 (89.4)
	Second-line immunotherapy agent	
	Nivolumab	6 (4.5)
	Pembrolizumab	5 (3.7)
	Third-line immunotherapy agent	
	Nivolumab	3 (2.2)
ECOG PS: Eastern Cooperative Oncology Group Performance Score		

*: Right colon (including transvers colon 2/3); **: Left colon (including transverse colon 1/3); ^&^ Xelox: Capecitabine + Oxaliplatin; ^&&^ Folfox: 5-Flurouracil + Leucoverin + Oxaliplatin; ^+^ Folfiri: 5-Flurouracil + Leucoverin + İrinotecan.

**Table 2:** Survival analysis of patients.

		PFS (Months)			OS (Months)					
		Univariate Analysis			Univariate Analysis			Multivariate Analysis		
		Median (Months)	%95 CI	*p*-Value	Median (Months)	%95 CI	*p*-Value	%95 CI	HR	*p*-Value
Age (year)				0.86			0.62			
	<60	9.9	8.4–11.3		44.9	25.0–64.7				
	≥60	12.5	9.8–15.1		44.6	17.4–71.8				
Sex				0.55						
	Female	11.1	7.5–14.7		32.6	6.7–58.5	0.59			
	Male	9.9	7.5–12.4		44.6	26.9–62.3				
Primary tumor location				0.045			0.036			
	Right	9.3	7.9–10.7		55.2	24.7–85.6				
	Left	12.5	9.1–15.8		44.9	16.0–73.7				
	Rectum	9.6	9.2–12.6		29.9	15.9–44.0				
De novo metastasis				0.55			0.04			
	Yes	10.9	9.2–12.6		72.2	21.1–68.1				
	No	10.3	61.1–14.4		54.5	11.8–53.9				
Mutation status				0.037			<0.01	2.72–20.05	7.9	<0.01
KRAS/NRAS mutant		11.5	6.5–16.4		50.8	20.4–69.3				
BRAF mutant		8.9	5.7–12.0		11.5	0.0–23.5				
RAS/BRAF wild		10.9	9.3–12.4		38.3	21.7–43.0				
Site of metastasis				0.092			0.053			
	Liver	11.1	8.4–13.8		50.8	24.6–77.1				
	Peritoneum	9.3	8.5–10.1		32.4	32.5–59.4				
	Others *	11.3	2.5–20.1		31.5	18.5–44.5				
Biological treatment				0.089						
	No	7.9	4.6–11.3							
	Bevacizumab	13.4	9.7–17.1							
	Cetuximab	12.5	10.2–14.7							
	Panitumumab	11.3	2.7–19.9							
Maintenance treatment				0.007			0.14	0.20–1.07	0.46	0.72
	Yes	14.0	12.4–15.5		55.2	29.9–80.4				
	No	8.7	7.1–10.3		31.5	8.9–54.2				
Maximal cytoreductive surgery				0.89			0.030	0.15–0.86	0.36	0.022
	Yes	8.9	6.8–11.0		55.2	15.0–50.2				
	No	11.1	9.3–13.0		32.6	17.9–92.4				
İmmunotherapy in subsequent treatment							0.005	0.28–0.51	0.12	0.005
	Yes				NR					
	No				31.5	20.2–42.8				

* Others: Lung, bone, brain, nonregional lap.

Median OS was 44 months (95% CI: 26.23–63.03) for all patients. No significant association was found between OS and clinicopathological features, such as age, gender, comorbidities, Ras mutation status, metastatic sites, and chemotherapy backbone as oxaliplatin- or irinotecan-based chemotherapy.

Median OS was 44.9 (95% CI: 16.06–73.73) months for the left colon, 55.2 (95% CI: 24.72–85.67) months for the right colon, and 29.9 (95% CI: 15.90–44.02) months for the rectum-localized tumors (*p*: 0.036). BRAF wild-type mCRC patients had a better OS. Median OS in BRAF wild type patients was 44.9 (95% CI: 19.1–70.6) months, while it was 11.5 (95% CI: 0.0–23.5) months (*p* < 0.001). Median OS in ‘de novo’ metastatic patients was 44.6 (95% CI: 21.11–68.15) months, and median OS in recurrent ones was 32.6 months (95% CI: 11.87–53.39) (*p*: 0.04). Median OS for the patients who received maintenance therapy was 55.2 months (95% CI: 29.91–80.49), and it was 31.56 (95% CI: 8.94–54.20) months for those who did not receive maintenance therapy. Although there was a numerical difference between the two groups, it did not reach a statistically significant level (*p*: 0.14). The patients with maximal cytoreduction had significantly higher OS than others who had no maximal cytoreductive surgery. Median OS was 55.2 months (95% CI: 17.99–92.40) versus 32.6 months (95% CI: 15.00–50.25) (*p*: 0.03). While median OS was not reached in patients receiving immunotherapy as a subsequent line, median OS was 31.5 months (95% CI: 20.26–42.86) in patients who did not receive any immunotherapy. The difference between the two groups was statistically significant (*p*: 0.005). In this study, six patients received nivolumab in the second-line setting, and five patients received pembrolizumab. After 32 months of follow-up, median progression-free survival was not reached. In the second-line setting, two patients had stable disease, and nine patients had partial regression. Three patients received nivolumab in the third-line setting. Two patients had partial regression, and one patient had stable disease.

**Figure 1:**
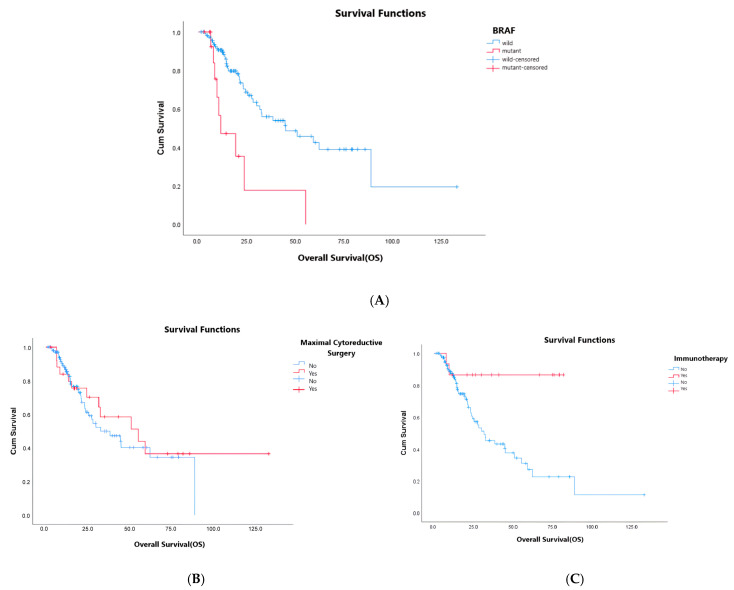
(**A**–**C**): OS analysis according to BRAF mutation status (**A**), maximal cytoreductive surgery (**B**), and subsequent immunotherapy (**C**).

In summary, tumor localization, BRAF mutation status, ‘de novo’ metastasis/recurrence disease, MCS, and subsequent IO seem to have prognostic value in univariate analysis (*p*: 0.036, *p* < 0.01, *p*: 0.04, *p*: 0.03, and *p*: 0.005, respectively). However, in multivariate analysis, mutation status, MCS, and subsequent IO are defined as prognostic factors for OS (*p* < 0.01, *p*: 0.022, and *p*: 0.005, respectively) (Figure 1). In multivariate analysis, OS was found to be statistically significantly lower in patients with BRAF mutation (HR: 7.9, 95% CI: 2.72–20.05, *p* < 0.01). Median OS was statistically significantly higher for the patients who underwent MCS (HR: 0.36, 95% CI: 0.15–0.86, *p*: 0.022) and others who received IO as a subsequent treatment (HR: 0.12, 95% CI: 0.28–0.51, *p*: 0.005).

## 4. Discussion

In this study, we retrospectively evaluated the efficacy of surgical and systemic treatments in metastatic dMMR/MSI-H colorectal cancer. We found that BRAF mutation, application of MCS, and the use of subsequent IO in patients who could not have received IO in the first-line setting were associated with OS.

The prognostic significance of ECOG-PS is well known in cancer. The patients with better ECOG-PS (0-1) cope with the systemic treatment with optimal dose density and intensity, leading to better outcomes. In our study, the majority of our patients in our study had ECOG PS (0-1). Therefore, we could not make a meaningful assessment according to ECOG-PS. The retrospective nature of our study and the heterogeneity of the patient population, consisting of ‘de novo’ metastatic and recurrent (metachronous) metastatic patients, were among the main limitations of our study. MSI-H metastatic colorectal cancer is a relatively rare group. But our study included only MSI-H mCRC patients with an acceptable number of cases for a meaningful statistical analysis.

When we evaluate the prognostic and predictive significance of mutation status in MSI-H mCRC patients on first-line targeted treatment, as anti-VEGF or anti-EGFRS, we determined a trend towards better numerically PFS, without statistical significance. Anti-EGFRs logically concluded to be noninferior to anti-VEGF in our study, though this retrospective study was not designed as a randomized noninferiority trial. However, our retrospective data were not consistent with the literature. The CALGB/SWOG 80,405 study, contrary to our results, showed that anti-VEGF treatment was superior to anti-EGFRs in MSI-H mCRC [17]. Hyper-selection may lead to bias for choosing targeted treatment agents in daily practice, and retrospective analysis of these data might have led to a contradiction here. In fact, microsatellite status is not a major determining factor for choosing the best targeted treatment drug.

BRAF mutation is a poor prognostic factor, and it is more common in right-sided colon cancer [18,19,20]. The BRAF mutation rate was 12.1% and the right colon localization rate was 61.4% in our study. These rates were similar to those in the literature [21]. However, all of our patients had MSI-H tumors, and our BRAF-mutant MSI-H mCRC patients had a worse prognosis than the others with BRAF wild-type ones. BRAF mutations are rarely detected in patients with oligometastatic colorectal cancer, potentially associated with aggressive tumor behavior [22]. In this study, MCS was found to be prognostic, and these patients were oligometastatic. Upon further examination, all of these patients were found to have wild-type BRAF mutations. The prognostic value of MCS is thought to be related to their BRAF wildness. More detailed studies, including molecular subtyping, are needed in the selection of patients who will undergo MCS.

Maximal cytoreductive surgery in metastatic cancer has been investigated in malignancies, including gastric cancer, breast cancer, and CRC [23,24,25,26]. In here, mesenchymal stem cells in the primary tumor are claimed to have a major role in the metastasis process, and this hypothesis contributed to the consideration of removal of primary tumor, almost in metastatic ones, to decrease the risk of progression [25]. However, surgical intervention in metastatic ones, especially with higher tumor loads, may also contribute to the progression via an increase in stress and cytokine release [27]. MCS has been shown to have better outcomes in ovarian serous carcinoma [28]. However, its contribution to survival has not been well documented in mCRC. In the review of 798 randomized and nonrandomized studies including 1086 patients, PTR failed to show any survival benefit in mCRC [29]. In contrast, it was determined that PTR improved OS independently in unresectable colorectal cancer in a retrospective observational study, including 1378 patients [30]. Additionally, the role of MCS in survival outcomes of MSI-H mCRC patients is unclear because of limited data due to the smaller number of patients in the literature. It is well known that MSI-H mCRC constitutes 3–5% of all mCRC cases. We consider that reduction in tumor volume via MCS, besides PTR, may contribute to better clinical outcomes according to the hypothesis mentioned above. Higher tumor load in MSI-H mCRC leads to higher immune antigen expression. It is an advantage for immunotherapy outcomes, but also a disadvantage for other systemic treatment modalities. In this study, we focused on the outcomes of first-line systemic treatment options, including targeted treatment rather than immunotherapy, in MSI-H mCRC. MCS was determined as a favorable prognostic factor in MSI-H mCRC patients. We believe that this area needs further evaluation in randomized clinical trials.

Immunotherapy is shown to have favorable outcomes in ‘immune hot’ tumors, including PDL1-overexpressed MSI-H ones. At first, we had encouraging data from the trials that evaluated the immunotherapy effect in later lines. KEYNOTE-164 and CheckMate 142 trial studies showed pembrolizumab’s and nivolumab’s efficacy in pretreated MSI-H mCRC patients, and both of them had FDA approval in second-line treatment of mCRC in 2017 [31,32]. Then, pembrolizumab became the standard of care in first-line therapy in MSI-H mCRC according to the results of the KEYNOTE-177 trial [11]. The CheckMate 8HW trial announced the first interim analysis of first-line nivolumab/ipilimumab combination immunotherapy with a significant PFS advantage over chemotherapy in dMMR/MSI-H mCRC patients last year [33]. Although immunotherapy is recommended in the first-line setting in dMMR/MSI-H mCRC in current guidelines, some patients cannot access it since it is not reimbursed in many countries, as is the case in our country. It is recommended as the first-line treatment strategy in these patients, and some of them may access it in later settings. There were 14 (10.6%) patients who had immunotherapy in later settings in our study, and this subgroup also had significantly better OS (*p*: 0.005). This real-life data supports the hypothesis that immunotherapy still works after targeted treatment (anti-VEGF or anti-EGFRs) in MSI-H mCRC.

Maintenance treatment is an option for selected patients in whom a clinical benefit has been achieved with a prior systemic treatment in mCRC. In this study, the PFS benefit with maintenance treatment following first-line treatment, including targeted treatment, has not been converted to OS benefit (*p*: 0.007, *p*: 0.72). It might have been related to the retrospective design of our study with a smaller number of patients.

The main limitation of this study was its retrospective nature. To maximize the number of patients, both de novo metastatic and metachronous metastatic patients were included. This limited the ability to interpret the study’s results. Prospective randomized controlled studies are needed on this subject. Furthermore, RAS mutations were assessed as either KRAS/NRAS mutations present or absent. Another limitation was the lack of knowledge of RAS mutation subtypes. In addition to these limitations, it demonstrated significant real-life outcomes in mCRC patients with MSI-H.

In conclusion, dMMR/MSI-H mCRC is a special entity in terms of tumor biology, prognosis, and treatment responses. We conclude that MSI-H mCRC patients with BRAF wild-type tumors, MCS, and subsequent IO had better survival with first-line targeted treatment compared to anti-VEGF or anti-EGFRs with 5FU/fluoropyrimidine-based chemotherapy. However, comprehensive randomized clinical trials are needed in this area.

The abstract of this study was presented as a poster at ASCO 2025 [34].

## Data Availability

The dataset of the study is available and can be requested from the responsible researcher if necessary.
